# Associations of leisure time physical activity volume, frequency, and intensity with all-cause mortality among cancer survivors

**DOI:** 10.1016/j.clinsp.2026.101113

**Published:** 2026-09-09

**Authors:** Yanxue Lian, Pincheng Luo, Linyi Zheng, Sha Li

**Affiliations:** a School of Medicine, University of Galway, Galway, Ireland; b Department of Public Physical Education, Hebei North University, Hebei, China

**Keywords:** Cancer survivors, Mortality, Physical activity, Survivorship care

## Abstract

**Purpose:**

To investigate whether the survival benefits of Leisure-Time Physical Activity (LTPA) among cancer survivors depend on total weekly volume, frequency of sessions, or proportion of vigorous-intensity activity.

**Methods:**

We analysed data from 3362 cancer survivors aged ≥ 20-years in the National Health and Nutrition Examination Survey (NHANES) 2007–2018, linked to mortality data through December 2019. LTPA was self-reported via the Global Physical Activity Questionnaire and categorized as inactive (< 150 min/week) or active (≥ 150 min/week). The active group was further stratified by session frequency (1‒2, 3‒4, ≥ 5 week) and vigorous-intensity proportion, defined as doubled vigorous minutes divided by total equivalent LTPA minutes (< 50%, ≥ 50%). Weighted Cox proportional hazards models estimated Hazard Ratios (HRs) for all-cause mortality, adjusting for sociodemographic, lifestyle, and clinical covariates.

**Results:**

Over a mean follow-up of 6.0-years, 856 deaths occurred. Meeting activity guidelines was associated with a 45% lower risk of all-cause mortality (HR = 0.55; 95% CI 0.43‒0.71). Among active participants, exploratory analyses suggested similar inverse associations across activity frequency categories and proportions of vigorous-intensity activity. Associations did not differ by age, sex, or marital status.

**Conclusion:**

Adherence to physical activity guidelines was associated with lower all-cause mortality among cancer survivors. Exploratory findings suggest that survival benefits may be achieved through a range of activity frequency and intensity patterns, supporting flexible approaches to physical activity promotion in survivorship care.

## Highlights

• Meeting the recommended weekly LTPA volume was associated with lower mortality.
• Frequency and intensity subgroup analyses were exploratory and underpowered.
• Findings support a cautious, volume-focused interpretation for survivorship care.

## Introduction

Physical activity confers numerous benefits for cancer survivors, including enhanced quality of life, improved psychological well-being with lower risks of anxiety and depression, improved muscle strength, and, importantly, reduced recurrence and mortality.^1^^,^^2^ These advantages have been observed across survivors of various cancers, including breast,^3^ pancreatic,^4^ ovarian,^5^ prostate,^6^ and colorectal cancers.^7^ Current guidelines recommend that survivors engage in at least 150-minutes of moderate-intensity activity, 75-minutes of vigorous-intensity activity, or an equivalent combination each week.^8^ Despite these recommendations, it remains uncertain whether the frequency of activity sessions or the intensity of activity plays a critical role in reducing mortality risk. Very few studies have directly examined the relationship between physical activity composition and survival in cancer patients, and existing findings remain inconclusive.^3^^,^^9^

Moreover, cancer survivors exhibit substantial heterogeneity in health status, time availability, and treatment-related side effects,^10^ which contribute to substantial differences in their composition of physical activity.^11^ For instance, survivors in relatively good health with ample free time may engage in vigorous-intensity exercise and complete multiple sessions per week, whereas those with work or caregiving responsibilities may be able to be active only on weekends. In contrast, more vulnerable survivors may be limited to low-to-moderate-intensity activity, requiring longer recovery periods, or may spread shorter bouts of activity across several days, with some days entirely reserved for rest. Additionally, even within the same individual, tolerance and responsiveness to exercise may fluctuate across different stages of the cancer trajectory, influenced by symptom severity and overall clinical condition.^8^ Therefore, understanding how specific activity compositions, including total volume, frequency, and intensity, relates to survival has important clinical and public health implications. Such evidence could inform the development of individualized exercise prescriptions that optimize survival outcomes and quality of life in cancer survivors. To address this knowledge gap, the present study examined the associations between Leisure-Time Physical Activity (LTPA) volume, frequency, and intensity composition, and all-cause mortality among cancer survivors.

## Methods

### Study design and population

**Fig. 1:**
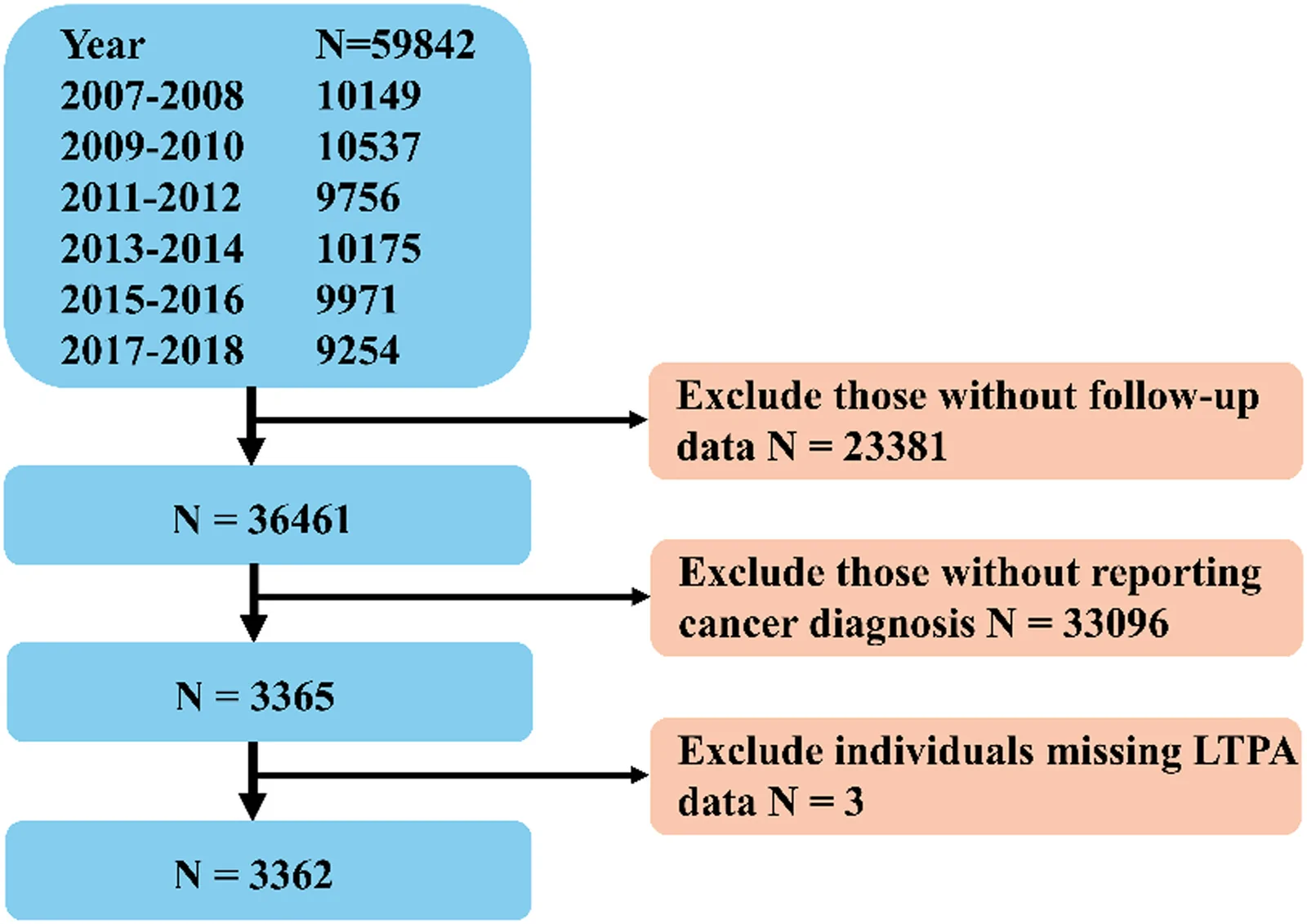
Flowchart of participant selection among U.S. adults aged ≥20-years in NHANES, 2007‒2018.

Data were obtained from the National Health and Nutrition Examination Survey (NHANES) 2007‒2018,^12^ a nationally representative survey linked to mortality data from the National Death Index through December 31, 2019.^13^ The survey was approved by the National Center for Health Statistics Research Ethics Review Board, and written informed consent was obtained from all participants. Cancer survivors aged ≥ 20-years with complete data on mortality status and LTPA were included. A total of 59,842 participants were initially identified. Of these, 23,381 individuals with available mortality status information were retained. Participants without a self-reported history of cancer or with missing data on LTPA were excluded. After applying these criteria, 3362 cancer survivors aged ≥ 20-years were included in the final analytic sample (Fig. 1). This study was reported in accordance with the Strengthening the Reporting of Observational Studies in Epidemiology (STROBE) guideline.

### Exposure (LTPA) and outcome (mortality)

LTPA was assessed using the Global Physical Activity Questionnaire, capturing the frequency (days/week) and duration (minutes/day) of moderate- and vigorous-intensity activity during a typical week. Total LTPA was calculated as moderate minutes plus twice the vigorous minutes per week. Participants were categorized as inactive (< 150 min/week) or active (≥ 150 min/week), based on current guidelines.^8^ The active group was further stratified by session frequency (1‒2, 3‒4, or ≥ 5 sessions/week) and by the proportion of vigorous-intensity activity (< 50%, or ≥ 50%), calculated as: (vigorous minutes *2) / (moderate minutes + vigorous minutes *2).

### Confounders

Confounders were selected based on prior literature and assessed concurrently with LTPA and were considered baseline variables. These included self-reported sociodemographic characteristics (age, sex, race, marital status, educational level, and family poverty income ratio). Poverty income ratio was categorized as high income (≥ 3.00), middle income (2.00‒2.99), near poor (1.00‒1.99), and poor (< 1.00).^14^ Lifestyle factors included smoking status (current, former, or never),^15^ alcohol consumption (≤ 1 drink, 2–14 drinks, or ≥ 15 drinks in the past 12-months),^16^ and body mass index (Body Mass Index [BMI], categorized as underweight, normal weight, overweight, or obesity).^17^ Chronic conditions included hypertension, diabetes, and cardiovascular disease, identified through self-report, clinical examination, and laboratory data. Hypertension was defined as a self-reported history of hypertension, use of antihypertensive medication, or measured systolic blood pressure ≥ 130 mm/Hg or diastolic blood pressure ≥ 80 mm/Hg.^18^ Diabetes was defined as a self-reported history of diabetes, use of hypoglycemic medication or insulin, fasting blood glucose ≥ 7.0 mmol/L, glycated hemoglobin ≥6.5%, or 2-hour postprandial glucose ≥ 11.0 mmol/L.^19^ Cardiovascular disease was self-reported and included myocardial infarction, coronary heart disease, congestive heart failure, angina, and stroke. The interval between cancer diagnosis and baseline assessment was also included as a covariate.^16^

### Statistical analysis

Weighted Cox proportional hazards models were used to estimate Hazard Ratios (HRs) and 95% Confidence Intervals (95% CIs) for all-cause mortality, with inactive participants as the reference group. Three sequential models were constructed with progressive adjustment for covariates. Model I was unadjusted; Model II was adjusted for sociodemographic characteristics; Model III was further adjusted for lifestyle factors, chronic conditions, and time interval between cancer diagnosis and baseline assessment. Missing data were handled using multiple chained imputation.

Several sensitivity analyses were conducted to evaluate the robustness of the findings. First, E-values were calculated for HRs estimates to quantify the minimum strength of association that an unmeasured confounder would need to have with both physical activity and mortality, conditional on the measured covariates, to fully explain away the observed associations.^20^ Second, participants with a BMI < 18.5 kg/m^2^ were excluded to reduce the potential influence of underlying illness, frailty, or disease-related weight loss. Third, participants with less than 12-months of follow-up were excluded to minimize potential reverse causation arising from pre-existing health conditions that may have limited physical activity before death. Finally, total LTPA duration (continuous, hours/week) was additionally included as a covariate in analyses examining physical activity frequency among active participants to determine whether the observed associations were independent of overall activity volume, given the potential correlation between activity frequency and total LTPA.

Moreover, subgroup analyses and interaction tests of the associations between total physical activity volume, frequency, and intensity composition and all-cause mortality among cancer survivors were conducted, stratified by age, sex, and marital status. In addition, participants were further stratified by cancer type, including prostate cancer, breast cancer, skin cancer, bladder cancer, colorectal cancer, blood cancer, lung cancer, lymphoma, melanoma, and other cancers. Within each cancer-specific subgroup, inactive participants (< 150 min/week of LTPA) served as the reference group, and HRs for all-cause mortality were estimated for active participants (≥150 min/week). However, due to the limited number of events within individual cancer types, further stratified analyses among active participants were not feasible to reliably examine the associations of physical activity frequency and vigorous physical activity ratio with mortality outcomes. Analyses were conducted using R, version 4.4.2 (R Foundation for Statistical Computing, Vienna, Austria). All statistical tests were two-sided, and a p-value < 0.05 was considered statistically significant.

## Results

A total of 3362 cancer survivors were included in the final analysis, of whom 906 (26.9%) were categorized in the physically active group (Table 1). Over a mean follow-up of 6.00 years, 25.5% of participants died. Compared with those who were physically inactive, physically active individuals were more likely to be younger, male, have a lower body mass index, higher education level, live with a companion, be never smokers, consume less alcohol, report fewer chronic conditions, and exhibit a lower mortality rate. The time interval between cancer diagnosis and baseline assessment was similar between the two groups.

**Table 1:** Sociodemographic characteristics of cancer survivors, NHANES 2007‒2018.

	Total	Inactive group	Active group
**N**	3362	2456	906
**Age (years), Mean ± SD**	65.67 ± 13.99	66.57 ± 13.50	63.25 ± 14.98
< 65	1328 (39.50%)	920 (37.46%)	408 (45.03%)
≥ 65	2034 (60.50%)	1536 (62.54%)	498 (54.97%)
**Sex, n (%)**			
Male	1594 (47.41%)	1109 (45.15%)	485 (53.53%)
Female	1768 (52.59%)	1347 (54.85%)	421 (46.47%)
**Race, n (%)**			
Mexican American	234 (6.96%)	187 (7.61%)	47 (5.19%)
Non-Hispanic White	2205 (65.59%)	1571 (63.97%)	634 (69.98%)
Non-Hispanic Black	496 (14.75%)	381 (15.51%)	115 (12.69%)
Other race	427 (12.70%)	317 (12.91%)	110 (12.14%)
**Education level (year), n (%)**			
< 9	315 (9.37%)	277 (11.28%)	38 (4.19%)
9‒12	1154 (34.32%)	947 (38.56%)	207 (22.85%)
> 12	1893 (56.31%)	1232 (50.16%)	661 (72.96%)
**Marital status, n (%)**			
Accompanied	1974 (58.72%)	1375 (55.99%)	599 (66.11%)
Living alone	1388 (41.28%)	1081 (44.01%)	307 (33.89%)
**Poverty Income Ratio, n (%)**			
Poor	540 (16.06%)	456 (18.57%)	84 (9.27%)
Near poor	912 (27.13%)	744 (30.29%)	168 (18.54%)
Middle income	539 (16.03%)	400 (16.29%)	139 (15.34%)
High income	1371 (40.78%)	856 (34.85%)	515 (56.84%)
**Body Mass index (kg/m2), Mean ± SD**	29.21 ± 6.57	29.62 ± 6.80	28.10 ± 5.76
< 18.5	51 (1.52%)	46 (1.87%)	5 (0.55%)
18.5‒24.9	845 (25.13%)	555 (22.60%)	290 (32.01%)
25‒29.9	1174 (34.92%)	836 (34.04%)	338 (37.31%)
≥ 30	1292 (38.43%)	1019 (41.49%)	273 (30.13%)
**Follow-up years, Mean ± SD**	6.00 ± 3.50	5.87 ± 3.54	6.33 ± 3.38
**Time interval between cancer diagnosis and baseline assessment (years)**	10.1 ± 10.5	10.1 ± 10.6	10.1 ± 10.3
**Smoking status, n (%)**			
Never smokers	1534 (45.63%)	1098 (44.71%)	436 (48.12%)
Ever smokers	1302 (38.73%)	924 (37.62%)	378 (41.72%)
Current smokers	526 (15.65%)	434 (17.67%)	92 (10.15%)
**Alcohol consumption, n (%)**			
Moderate drinker	2286 (68.00%)	1648 (67.10%)	638 (70.42%)
Heavier drinker	1076 (32.00%)	808 (32.90%)	268 (29.58%)
**Cardiovascular diseases, n (%)**			
Yes	826 (24.57%)	673 (27.40%)	153 (16.89%)
No	2536 (75.43%)	1783 (72.60%)	753 (83.11%)
**Hypertension, n (%)**			
Yes	2603 (77.42%)	1947 (79.28%)	656 (72.41%)
No	759 (22.58%)	509 (20.72%)	250 (27.59%)
**Diabetes, n (%)**			
Yes	925 (27.51%)	752 (30.62%)	173 (19.09%)
No	2437 (72.49%)	1704 (69.38%)	733 (80.91%)
**Mortality, n (%)**			
Alive	2506 (74.54%)	1733 (70.56%)	773 (85.32%)
Deceased	856 (25.46%)	723 (29.44%)	133 (14.68%)

Note: Continuous variables are presented as mean ± SD, and categorical variables as number and percentage (n, %). SD, Standard Deviation.

In fully adjusted models, engaging in ≥ 150 minutes of physical activity per week was associated with a 45% lower risk of all-cause mortality compared with inactivity (HR = 0.55; 95% CI 0.43‒0.71). In exploratory stratified analyses, survival advantages were observed across various activity distributions: those concentrating activity into 1 to 2 sessions per week had an HR of 0.50 (95% CI 0.26‒0.97), those engaging in 3 to 4 sessions per week had an HR of 0.60 (95% CI 0.41‒0.89), and those active on ≥ 5 days per week had an HR of 0.53 (95% CI 0.40‒0.71). These associations remained consistent across different proportions of vigorous-intensity activity: for participants with < 50% of activity as vigorous, the HR was 0.61 (95% CI 0.48‒0.78), and for those with ≥ 50% vigorous activity, the HR was 0.37 (95% CI 0.17‒0.78) (Table 2).

**Table 2:** Associations of total physical activity volume, frequency, and intensity composition with all-cause mortality among cancer survivors, weighted.

	N (cases)	Model I	Model II	Model III
**Inactive group** (< 150 min/week)	2456 (723)	1 [reference]	1 [reference]	1 [reference]
**Active group** (≥ 150 min/week)	906 (133)	0.44 (0.34–0.56)	0.52 (0.40–0.67)	0.55 (0.43–0.71)
**Further stratification: Active group by frequency**^a^				
1‒2 sessions/week	134 (16)	0.36 (0.18–0.76)	0.47 (0.23–0.95)	0.50 (0.26–0.97)
3‒4 sessions/week	356 (49)	0.44 (0.30–0.66)	0.56 (0.38–0.83)	0.60 (0.41–0.89)
≥ 5 sessions/week	416 (68)	0.45 (0.34–0.59)	0.51 (0.38–0.68)	0.53 (0.40–0.71)
**Further stratification: Active group by vigorous physical activity ratio**^a^				
< 50%	603 (106)	0.57 (0.43–0.76)	0.60 (0.47–0.77)	0.61 (0.48–0.78)
≥ 50%	303 (27)	0.19 (0.08–0.49)	0.30 (0.14–0.65)	0.37 (0.17–0.78)

Note: Unadjusted Model I; Model II adjusted for sociodemographic characteristics (age, sex, race, marital status, education level, PIR); Model III further adjusted for lifestyle factors (BMI, smoking status, alcohol consumption), chronic conditions (hypertension, diabetes, and cardiovascular disease), and time interval between cancer diagnosis and baseline assessment.

a Effect estimates and confidence intervals should be interpreted with caution due to the limited number of events and relatively small sample sizes.

E-value analyses showed that the point estimates ranged from 2.66 to 4.85 across physical activity exposure metrics (Online Resource, Table S1), indicating that an unmeasured confounder would need to be associated with both physical activity patterns and mortality by a risk ratio of approximately 3–5-fold to fully explain away the observed associations, consistent with moderate robustness to unmeasured confounding. The findings from the primary analyses remained materially unchanged in sensitivity analyses, including exclusion of participants who were underweight (BMI < 18.5 kg/m^2^; Online Resource, Table S2), exclusion of those with less than 12-months of follow-up (Online Resource, Table S3), and additional adjustment for total LTPA duration in analyses of activity frequency (Online Resource, Table S4).

Furthermore, in subgroup analyses stratified by age (< 65 vs. ≥ 65 years, a cut-off selected to reflect the conventional retirement age, which marks a significant transition in “free time” availability and baseline functional status), sex (male vs. female), and marital status (married vs. unmarried), achieving the recommended total physical activity volume (≥ 150 min/week) was consistently associated with lower all-cause mortality. Notably, the associations between physical activity frequency and intensity composition and all-cause mortality were not significantly modified by age, sex, or marital status (Online Resource, Tables S5‒S7).

In analyses stratified by cancer type, significant inverse associations between meeting physical activity guidelines (≥ 150 min/week) and all-cause mortality were observed among participants with prostate cancer, breast cancer, and other cancers (Online Resource, Table S8). No statistically significant associations were detected in the remaining cancer subgroups, which may be attributable to limited statistical power due to small sample sizes and a low number of events within individual strata.

## Discussion

In this nationally representative cohort of cancer survivors, meeting the recommended threshold of ≥ 150 minutes of physical activity per week was associated with a substantially lower risk of all-cause mortality. Similar associations were observed when examining categories of weekly activity frequency and proportions of vigorous-intensity activity, although these subgroup analyses were exploratory. These findings should therefore be interpreted with caution. While they may suggest practical flexibility in how survivors accumulate physical activity, they do not establish that different frequency or intensity patterns confer equivalent survival benefits. These findings extend current evidence on physical activity patterns and survival in cancer survivors.

Current physical activity guidelines for the general population recommend at least 150 minutes of moderate-intensity or 75 minutes of vigorous-intensity aerobic activity per week.^8^ The present findings suggest that this recommendation may also be applicable to cancer survivors in relation to survival. This is supported by prior evidence: a meta-analysis of 151 cohorts including nearly 1.5 million cancer patients reported that post-diagnosis physical activity was associated with a 24%‒31% reduction in cancer-specific mortality, depending on cancer type.^21^ Similarly, a randomized trial in colon cancer survivors demonstrated that overall survival over a median follow-up of 7.9 years was significantly longer in a structured exercise group compared with a health-education control group.^22^ Higher post-diagnosis physical activity has also been linked to lower risk of recurrence; a review of 15,298 patients found that greater self-reported activity volumes were associated with a 45% reduction in recurrence risk (HR = 0.65, 95% CI 0.56‒0.75).^23^

Although well-documented benefits, current guidelines offer limited specificity regarding the optimal distribution of exercise frequency and intensity. In our study, lower mortality risk was observed across subgroups defined by varying frequency and proportions of vigorous-intensity activity. This finding is supported by a previous meta-analysis, which showed that accumulating ≥150 minutes of moderate-to-vigorous physical activity within 1‒2 days or across ≥3 days yields similar benefits across multiple health outcomes.^24^ In addition, clinical trial evidence has demonstrated no meaningful differences in outcomes between high-intensity and low-to-moderate-intensity exercise during cancer treatment.^9^ The overarching outcomes suggest that total accumulated weekly activity volume is robustly associated with the observed survival benefit. While the present data emphasize the importance of achieving total volume targets, the underpowered nature of the stratified comparisons precludes drawing strong conclusions regarding the irrelevance of specific exercise frequencies or intensity proportions.

Clinically, these findings shift attention from strict compliance with precise exercise “doses” to a more comprehensive “volume-first” counselling approach. For survivors with limited functional capacity or significant treatment-related sequelae, such as peripheral neuropathy,^25^ lymphedema,^26^ or severe cancer-related fatigue,^10^ traditional frequent or vigorous sessions are often prohibitive. The current results provide a reassuring data basis for clinicians to guide these patients: survival benefits may be attained by accumulating target volume through low-to-moderate intensity sessions distributed throughout the week, or even focused into fewer sessions as tolerated.

Moreover, the baseline data indicating that physically active adults frequently had higher educational levels highlights how socioeconomic considerations may impede the practicality of achieving stringent activity objectives. Thereby, an adaptable strategy involves “engaging survivors at their current state” by advocating for practical, accessible activities (e.g., home-based or unstructured movement) to alleviate these socioeconomic limitations.^27^ This flexibility directly addresses prevalent obstacles to exercise in oncology, perhaps alleviating the psychological strain and “failure perception” linked to the inability to achieve more rigorous, high-frequency activity objectives.

Several biological mechanisms may underlie the observed protective associations between physical activity and mortality among cancer survivors. Regular physical activity is known to enhance insulin sensitivity,^28^ mediate sex hormones,^29^ reduce systemic inflammation,^30^ and emerging evidence highlights additional exercise–oncology crosstalk. Specifically, exercise-induced myokines exert direct anti-tumor effects,^31^ and physical activity favorably reprograms the tumor microenvironment by enhancing cytotoxic immune cell infiltration.^32^ Additionally, physical activity can improve cardiorespiratory fitness, preserve lean muscle mass, alleviate fatigue, and enhance psychological well-being, factors that collectively contribute to better survival outcomes.^33^ Whether these mechanisms differ based on activity frequency or intensity remains unclear and should be examined in future mechanistic studies.

Nevertheless, the observed association between achieving guideline-level LTPA and lower all-cause mortality has significant implications for the formulation of Survivorship Care Plans (SCPs). Whereas population-level guidelines establish essential public health targets, they frequently lack the specificity needed for personalized exercise oncology. This study emphasizes a crucial differentiation: population recommendations act as a “target”, while individualized prescriptions must operate as a “pathway” customized to a survivor's specific clinical course and lifestyle limitations. In clinical counselling, the primary emphasis should be placed on helping survivors achieve a feasible total weekly activity volume, while tailoring frequency and intensity to individual tolerance, functional capacity, and treatment-related limitations. This volume-focused approach may improve the feasibility, adherence, and inclusivity of physical activity promotion in survivorship care, without implying that specific frequency or intensity patterns confer equivalent survival benefits. Nonetheless, physical activity capacity and survival prognosis may vary substantially according to tumor burden, disease stage, and treatment-related functional limitations.

This study is among the few that employ a substantial, nationally representative sample to analyze the intricate composition of physical activity, including volume, frequency, and intensity, in cancer survivors. Despite these strengths, several limitations should be acknowledged. First, physical activity was self-reported, which may introduce recall bias or misclassification. Although we used a validated instrument and adjusted for a wide range of sociodemographic, clinical, and lifestyle covariates, residual confounding from unmeasured factors, particularly functional status and treatment-related characteristics, cannot be excluded. Thus, the observed associations should be interpreted as supportive population-level evidence rather than definitive evidence that physical activity confers equivalent survival benefits across different disease stages or treatment contexts. Second, although we conducted subgroup analyses across major cancer types, limited sample sizes and event numbers precluded further stratification of physically active participants by activity frequency and intensity composition. In addition, substantial heterogeneity likely remained within cancer-specific subgroups, as NHANES does not provide detailed information on cancer stage, disease severity, or treatment history, which may influence both physical activity participation and mortality risk. Third, the relatively small sample sizes and limited number of events in some subgroup analyses may have reduced statistical power, and the corresponding estimates should therefore be interpreted cautiously. Finally, as this is an observational study, causal inferences should be made cautiously. Reverse causation remains a possibility, although sensitivity analyses yielded consistent results and E-value estimates (2.66‒4.85) suggested moderate robustness to unmeasured confounding, helping to mitigate this concern.

## Conclusion

In conclusion, adherence to physical activity guidelines was associated with lower all-cause mortality among cancer survivors. Flexible distributions of activity frequency and vigorous-intensity proportions might also confer similar benefits, although the statistical precision of these subgroups needs careful interpretation. Thus, these findings highlight the feasibility of flexible activity schedules rather than definitively dismissing the potential importance of exercise intensity or frequency. Given the heterogeneity of cancer types and survivorship trajectories, these findings should be interpreted as population-level evidence and should not replace tumor-specific exercise recommendations. Ultimately, the evidence strongly supports the adoption of adaptable, individualized approaches to physical activity promotion in survivorship care, contributing to more inclusive and pragmatic physical activity recommendations for the growing population of cancer survivors.

## Authors’ contributions

Yanxue Lian and Pincheng Luo contributed equally to this work. Yanxue Lian and Pincheng Luo: Conceptualization, Methodology, Software, Validation, Formal analysis, Investigation, Visualization, Writing-original draft, and Writing-review & editing. Linyi Zheng: Writing-original draft, and Writing-review & editing. Sha Li: Conceptualization, Methodology, Validation, Visualization, Supervision, Writing-original draft, and Writing-review & editing. All authors have read and approved the final manuscript.

## Ethics declarations

This study was conducted using publicly available data from the National Health and Nutrition Examination Survey (NHANES). NHANES data are de-identified and freely accessible to researchers. Therefore, no additional institutional ethical approval or informed consent was required. The study was performed in accordance with the principles of the Declaration of Helsinki, and data use complied with the NHANES data usage guidelines.

## Generative AI and AI-assisted technologies

Not applicable.

## Funding

The author(s) received no specific funding for this work.

## Conflicts of interest

The authors declare no conflicts of interest.

## Data Availability

The datasets analyzed in this study are publicly available from the National Health and Nutrition Examination Survey (NHANES), National Center for Health Statistics, Centers for Disease Control and Prevention, at: https://wwwn.cdc.gov/nchs/nhanes/. The linked mortality data are publicly available from the National Center for Health Statistics Linked Mortality Files at: https://www.cdc.gov/nchs/linked-data/mortality-files/index.html. The processed datasets, extraction tables, and analysis code generated during the current study are available from the corresponding author upon reasonable request.

## Appendix Supplementary materials

Image, application 1
