# Supplementary material for: Associations of leisure time physical activity volume, frequency, and intensity with all-cause mortality among cancer survivors
Source: Clinics (Sao Paulo). 2026 Sep 9;81:101113. doi: 10.1016/j.clinsp.2026.101113 (PMC13573692; doi:10.1016/j.clinsp.2026.101113)
Supplement: Supplementary file 1 [file mmc1.docx]

CLINICS-D-26-00709_Supplementary Material

**Table S1** Multivariable-adjusted hazard ratios and E-values for unmeasured confounding analysis based on the fully adjusted model.

|  | **Model III HR (95 CI%)** | **E-value (point estimate)** | **E-value (CI limit closest to null)** |
| --- | --- | --- | --- |
| **Inactive group** (< 150 min/week) | 1 [reference] | NA | NA |
| **Active group** (≥ 150 min/week) | 0.55 (0.43–0.71) | 3.04 | 2.17 |
| **Further stratification: Active group by frequency**^a^ | | | |
| 1-2 sessions/week | 0.50 (0.26–0.97) | 3.41 | 1.21 |
| 3-4 sessions/week | 0.60 (0.41–0.89) | 2.72 | 1.50 |
| ≥ 5 sessions/week | 0.53 (0.40–0.71) | 3.18 | 2.17 |
| **Further stratification: Active group by vigorous physical activity ratio**^a^ | | | |
| < 50% | 0.61 (0.48–0.78) | 2.66 | 1.88 |
| ≥ 50% | 0.37 (0.17–0.78) | 4.85 | 1.88 |

Note: Model III fully adjusted for sociodemographic characteristics (age, sex, race, marital status, education level, PIR), lifestyle factors (BMI, smoking status, alcohol consumption), chronic conditions (hypertension, diabetes, and cardiovascular disease), and time interval between cancer diagnosis and baseline assessment.

^a^Effect estimates and confidence intervals should be interpreted with caution due to the limited number of events and relatively small sample sizes.

**Table S2** Sensitivity analysis of the associations between total physical activity volume, frequency, and intensity composition and all-cause mortality among cancer survivors after excluding individuals with BMI < 18.5 kg/m^2^.

|  | **N (cases)** | **Full adjusted model** |
| --- | --- | --- |
| **Inactive group** (< 150 min/week) | 2410 (701) | 1 [reference] |
| **Active group** (≥ 150 min/week) | 901 (132) | 0.59 (0.44–0.72) |
| **Further stratification: Active group by frequency^a^** | | |
| 1‒2 sessions/week | 134 (16) | 0.50 (0.26–0.96) |
| 3‒4 sessions/week | 354 (49) | 0.60 (0.41–0.90) |
| ≥ 5 sessions/week | 413 (67) | 0.55 (0.41–0.73) |
| **Further stratification: Active group by vigorous physical activity ratio^a^** | | |
| 0 | 558 (103) | 0.65 (0.51–0.84) |
| < 50% | 42 (2) | 0.17 (0.04–0.77) |
| ≥ 50% | 301 (27) | 0.35 (0.21–0.58) |

Note: The model fully adjusted for sociodemographic characteristics (age, sex, race, marital status, education level, PIR), lifestyle factors (BMI, smoking status, alcohol consumption), chronic conditions (hypertension, diabetes, and cardiovascular disease), and time interval between cancer diagnosis and baseline assessment

^a^Effect estimates and confidence intervals should be interpreted with caution due to the limited number of events and relatively small sample sizes.

**Table S3** Sensitivity analysis of the associations between total physical activity volume, frequency, and intensity composition and all-cause mortality among cancer survivors after excluding those with less than 12-months of follow-up.

|  | **N (cases)** | **Full adjusted model** |
| --- | --- | --- |
| **Inactive group** (< 150 min/week) | 2361 (628) | 1 [reference] |
| **Active group** (≥ 150 min/week) | 899 (126) | 0.59 (0.45–0.78) |
| **Further stratification: Active group by frequency^a^** | | |
| 1‒2 sessions/week | 132 (14) | 0.46 (0.22–0.95) |
| 3‒4 sessions/week | 353 (46) | 0.62 (0.42–0.93) |
| ≥ 5 sessions/week | 414 (66) | 0.55 (0.40–0.74) |
| **Further stratification: Active group by vigorous physical activity ratio^a^** | | |
| 0 | 556 (99) | 0.69 (0.52–0.90) |
| < 50% | 301 (2) | 0.18 (0.04–0.86) |
| ≥ 50% | 42 (25) | 0.36 (0.21–0.64) |

Note: The model fully adjusted for sociodemographic characteristics (age, sex, race, marital status, education level, PIR), lifestyle factors (BMI, smoking status, alcohol consumption), chronic conditions (hypertension, diabetes, and cardiovascular disease), and time interval between cancer diagnosis and baseline assessment

^a^Effect estimates and confidence intervals should be interpreted with caution due to the limited number of events and relatively small sample sizes.

**Table S4** Sensitivity analysis of the associations between physical activity frequency and all-cause mortality among cancer survivors, with additional adjustment for total leisure-time physical activity duration.

|  | **N (cases)** | **Full adjusted model** |
| --- | --- | --- |
| **Inactive group** (< 150 min/week) | 2456 (723) | 1 [reference] |
| **Further stratification: Active group by frequency^a^** | | |
| 1‒2 sessions/week | 132 (16) | 0.53 (0.30–0.95) |
| 3‒4 sessions/week | 353 (49) | 0.61 (0.43–0.86) |
| ≥ 5 sessions/week | 414 (68) | 0.57 (0.40–0.82) |

Note: The model fully adjusted for sociodemographic characteristics (age, sex, race, marital status, education level, PIR), lifestyle factors (BMI, smoking status, alcohol consumption), chronic conditions (hypertension, diabetes, and cardiovascular disease), and time interval between cancer diagnosis and baseline assessment, with additional adjustment for total leisure-time physical activity duration.

^a^Effect estimates and confidence intervals should be interpreted with caution due to the limited number of events and relatively small sample sizes.

**Table S5** Subgroup analyses and interaction tests of the associations between total physical activity volume, frequency, and intensity composition and all-cause mortality among cancer survivors, stratified by age.

|  |  | **< 65** | | **≥ 65** | | **p for interaction** |
| --- | --- | --- | --- | --- | --- | --- |
|  |  | **N (cases)** | **HR (95 CI%)** | **N (cases)** | **HR (95 CI%)** |  |
|  | Inactive group (< 150 min/week) | 920 (118) | 1 [reference] | 1536 (605) | 1 [reference] | 0.96 |
|  | Active group (≥ 150 min/week) | 408 (20) | 0.61 (0.35–1.07) | 498 (113) | 0.53 (0.40–0.69) |  |
| **Further stratification: Active group by frequency^a^** |  |  |  |  |  | 0.55 |
|  | 1‒2 sessions/week | 68 (2) | 0.15 (0.02–1.38) | 66 (14) | 0.62 (0.28–1.38) |  |
|  | 3‒4 sessions/week | 170 (9) | 0.52 (0.18–1.51) | 186 (40) | 0.59 (0.42–0.83) |  |
|  | ≥ 5 sessions/week | 170 (9) | 0.91 (0.45–1.83) | 246 (59) | 0.45 (0.30–0.69) |  |
| **Further stratification: Active group by vigorous physical activity ratio^a^** |  |  |  |  |  | 0.71 |
|  | 0 | 204 (14) | 0.87 (0.49–1.57) | 357 (90) | 0.57 (0.43–0.77) |  |
|  | < 50% | 26 (1) | 0.15 (0.02–1.17) | 16 (1) | 0.15 (0.02–1.12) |  |
|  | ≥ 50% | 178 (5) | 0.23 (0.07–0.73) | 125 (22) | 0.41 (0.25–0.66) |  |

Note: The model fully adjusted for sociodemographic characteristics (sex, race, marital status, education level, PIR), lifestyle factors (BMI, smoking status, alcohol consumption), chronic conditions (hypertension, diabetes, and cardiovascular disease), and time interval between cancer diagnosis and baseline assessment

^a^Effect estimates and confidence intervals should be interpreted with caution due to the limited number of events and relatively small sample sizes.

**Table S6** Subgroup analyses and interaction tests of the associations between total physical activity volume, frequency, and intensity composition and all-cause mortality among cancer survivors, stratified by sex.

|  |  | **Male** | | **Female** | | **p for interaction** |
| --- | --- | --- | --- | --- | --- | --- |
|  |  | **N (cases)** | **HR (95 CI%)** | **N (cases)** | **HR (95 CI%)** |  |
|  | Inactive group (< 150 min/week) | 1109 (410) | 1 [reference] | 1347 (313) | 1 [reference] | 0.29 |
|  | Active group (≥ 150 min/week) | 485 (97) | 0.59 (0.42–0.83) | 421 (36) | 0.47 (0.28–0.79) |  |
| **Further stratification: Active group by frequency**^a^ |  |  |  |  |  | 0.81 |
|  | 1‒2 sessions/week | 90 (15) | 0.57 (0.30–1.11) | 44 (1) | 0.11 (0.01–0.89) |  |
|  | 3‒4 sessions/week | 178 (32) | 0.59 (0.36–0.96) | 178 (17) | 0.56 (0.28–1.13) |  |
|  | ≥ 5 sessions/week | 217 (50) | 0.60 (0.39–0.93) | 199 (18) | 0.45 (0.21–0.94) |  |
| **Further stratification: Active group by vigorous physical activity ratio**^a^ |  |  |  |  |  | 0.40 |
|  | < 50% | 324 (75) | 0.68 (0.47–0.98) | 279 (31) | 0.53 (0.33–0.86) |  |
|  | ≥ 50% | 161 (22) | 0.36 (0.14–0.93) | 142 (5) | 0.25 (0.02–2.70) |  |

Note: The model fully adjusted for sociodemographic characteristics (age, race, marital status, education level, PIR), lifestyle factors (BMI, smoking status, alcohol consumption), chronic conditions (hypertension, diabetes, and cardiovascular disease), and time interval between cancer diagnosis and baseline assessment.

^a^ Effect estimates and confidence intervals should be interpreted with caution due to the limited number of events and relatively small sample sizes.

**Table S7** Subgroup analyses and interaction tests of the associations between total physical activity volume, frequency, and intensity composition and all-cause mortality among cancer survivors, stratified by marital status.

|  |  | **Accompanied** | | **Alone** | | **p for interaction** |
| --- | --- | --- | --- | --- | --- | --- |
|  |  | **N (cases)** | **HR (95 CI%)** | **N (cases)** | **HR (95 CI%)** |  |
|  | Inactive group (< 150 min/week) | 1375 (356) | 1 [reference] | 1081 (367) | 1 [reference] | 0.70 |
|  | Active group (≥ 150 min/week) | 599 (71) | 0.54 (0.37–0.81) | 307 (62) | 0.58 (0.37–0.93) |  |
| **Further stratification: Active group by frequency**^a^ |  |  |  |  |  | 0.84 |
|  | 1‒2 sessions/week | 89 (8) | 0.56 (0.27–1.18) | 45 (8) | 0.38 (0.03–4.77) |  |
|  | 3‒4 sessions/week | 241 (26) | 0.55 (0.27–1.09) | 115 (23) | 0.69 (0.38–1.25) |  |
|  | ≥ 5 sessions/week | 269 (37) | 0.54 (0.33–0.88) | 147 (31) | 0.56 (0.31–1.00) |  |
| **Further stratification: Active group by vigorous physical activity ratio**^a^ |  |  |  |  |  | 0.71 |
|  | < 50% | 403 (62) | 0.63 (0.44–0.91) | 200 (44) | 0.64 (0.38–1.07) |  |
|  | ≥ 50% | 196 (9) | 0.20 (0.02–1.85) | 107 (18) | 0.47 (0.25–0.91) |  |

Note: The model fully adjusted for sociodemographic characteristics (age, gender, race, education level, PIR), lifestyle factors (BMI, smoking status, alcohol consumption), chronic conditions (hypertension, diabetes, and cardiovascular disease), and time interval between cancer diagnosis and baseline assessment.

^a^ Effect estimates and confidence intervals should be interpreted with caution due to the limited number of events and relatively small sample sizes.

**Table S8** Cancer-specific hazard ratios for all-cause mortality associated with meeting physical activity guidelines (≥150 min/week), with inactive participants as the reference group.

| **Cancer types** | **Total (deaths)** | **Inactive cases (deaths)** | **Active cases (deaths)** | **Fully adjusted model HR (95 CI%)** |
| --- | --- | --- | --- | --- |
| Prostate cancer | 513 (150) | 362 (124) | 151 (26) | 0.38 (0.21–0.69) |
| Breast cancer | 518 (118) | 404 (106) | 114 (12) | 0.31 (0.12–0.79) |
| Skin cancer | 735 (178) | 493 (140) | 242 (38) | 0.73 (0.34–1.57) |
| Bladder | 83 (31) | 64 (29) | 19 (2) | 0.18 (0.01–3.47) |
| Colorectal cancer | 238 (88) | 191 (73) | 47 (15) | 0.60 (0.30–1.20) |
| Blood cancer | 46 (15) | 35 (13) | 11 (2) | 0.69 (0.21–2.26) |
| Lung cancer | 82 (38) | 70 (35) | 12 (3) | 0.74 (0.27–2.08) |
| lymphoma | 76 (21) | 50 (17) | 26 (4) | 0.33 (0.04–2.52) |
| Melanoma | 195 (41) | 129 (28) | 66 (13) | 0.98 (0.41–2.36) |
| Other cancers | 1125 (249) | 840 (213) | 285 (36) | 0.46 (0.28–0.77) |

Note: The model fully adjusted for sociodemographic characteristics (age, gender, race, education level, PIR), lifestyle factors (BMI, smoking status, alcohol consumption), chronic conditions (hypertension, diabetes, and cardiovascular disease), and time interval between cancer diagnosis and baseline assessment.
